# Bulk Segregant Analysis Using Single Nucleotide Polymorphism Microarrays

**DOI:** 10.1371/journal.pone.0015993

**Published:** 2011-01-27

**Authors:** Anthony Becker, Dai-Yin Chao, Xu Zhang, David E. Salt, Ivan Baxter

**Affiliations:** 1 Donald Danforth Plant Sciences Center, St. Louis, Missouri, United States of America; 2 Center for Plant Environmental Stress Physiology, Purdue University, West Lafayette, Indiana, United States of America; 3 Department of Genetics, University of Chicago, Chicago, Illinois, United States of America; 4 Plant Genetics Research Unit, United States Department of Agriculture-Agricutlural Research Service, St. Louis, Missouri, United States of America; Cairo University, Egypt

## Abstract

Bulk segregant analysis (BSA) using microarrays, and extreme array mapping (XAM) have recently been used to rapidly identify genomic regions associated with phenotypes in multiple species. These experiments, however, require the identification of single feature polymorphisms (SFP) between the cross parents for each new combination of genotypes, which raises the cost of experiments. The availability of the genomic polymorphism data in *Arabidopsis thaliana*, coupled with the efficient designs of Single Nucleotide Polymorphism (SNP) genotyping arrays removes the requirement for SFP detection and lowers the per array cost, thereby lowering the overall cost per experiment. To demonstrate that these approaches would be functional on SNP arrays and determine confidence intervals, we analyzed hybridizations of natural accessions to the Arabidopsis ATSNPTILE array and simulated BSA or XAM given a variety of gene models, populations, and bulk selection parameters. Our results show a striking degree of correlation between the genotyping output of both methods, which suggests that the benefit of SFP genotyping in context of BSA can be had with the cheaper, more efficient SNP arrays. As a final proof of concept, we hybridized the DNA from bulks of an F2 mapping population of a Sulfur and Selenium ionomics mutant to both the Arabidopsis ATTILE1R and ATSNPTILE arrays, which produced almost identical results. We have produced R scripts that prompt the user for the required parameters and perform the BSA analysis using the ATSNPTILE1 array and have provided them as supplemental data files.

## Introduction

Mapping the causal allele or alleles for a particular trait is one of the most common methods for learning about the genetic processes underlying biological function. One method to rapidly identify markers in a genomic region linked to a phenotype is Bulk Segregant Analysis (BSA) [1]. BSA partitions a population from a single cross into two pools, or bulks, according to a single trait, so that each bulk contains individuals corresponding to a particular phenotype or specific section of a phenotypic range. The method uses marker measurements of pooled genomic DNA samples from each bulk to measure correlation between marker and phenotype and thereby designate a probable location for the gene basedon that correlation. BSA was first combined with microarray genotyping in yeast using arrays designed for measuring mRNA expression [2]. The technique allows for the parallel interrogation of thousands of single feature polymorphisms (SFPs), i.e. differences in the binding intensity to a particular oligonucleotide probe between two different samples of genomic DNA. Later studies showed the potential of BSA combined with SFP genotyping arrays in successfully mapping genes to mutant phenotypes in more complex genomes, such as Arabidopsis [3], [4] and the technique has been used to map mutants in several species [5]–[10].

The microarray genotyping approach was later extended to BSA-based investigations of quantitative traits where the pools were selected from the extreme ends of phenotypes in a continuously variable population. This process, called eXtreme Array Mapping (XAM), has successfully mapped Quantitative Trait Loci (QTL) in Arabidopsis and offers a time-efficient and cost-effective method of discovering new QTL [11]. However, SFP marker approaches require multiple parental hybridizations for permutation testing to identify viable SFPs among the features on a given array [3], [11]. This method of identifying markers can be expensive given the low percentage of features that qualify as viable markers.

Recently, arrays that are designed to specifically probe known single nucleotide polymorphisms (SNPs), using probes for both alleles, have been developed for multiple species. SNP genotyping eliminates the need to identify viable marker features via permutation testing, relying instead on SNPs identified by genome resequencing methods. In the case of Arabidopiss, a SNP array was constructed with the ability to interrogate over 200,000 SNPs and this array has been used to genotype over 1,000 natural accessions [12], [13 and unpublished data from the Nordborg and Borevitz labs]. These resources provide thousands of SNP markers between almost any two lines of the thousands of Arabidopsis (closely related lines will have fewer SNPs). In addition to this advantage, recent reductions in feature redundancy of SNP genotyping arrays have made SNP arrays more data and cost efficient, reducing the number of features per SNP from up to 40 probes to only 4 probes [14]. Thus, the price and effort spent per viable marker on a SNP genotyping array is significantly lower than the price and effort spent on a SFP array, making SNP genotyping a clear improvement over SFP genotyping for use with BSA and XAM.

However, like SFP genotyping arrays, even after preprocessing, SNP arrays produce a considerable amount of noise in their intensity readings due to variation in probe binding strength, random and nonspecific binding. This noise can blur both the intensity and position of the peak signal that represents the probable location of the mapped gene. In this study, we have developed a platform for performing and simulating BSA and XAM with microarrays in several gene models, using the preferred SNP genotyping arrays for Arabidopsis. Our method is easily adaptable for use with SNP arrays for other species.

## Materials and Methods

### Arrays

The Affymetrix 250K ATSNPTILE1 array interrogates ∼250,000 SNPs in the *Arabidopsis* genome with Col-0 as a reference genotype. Atwell *et al*. [12] genotyped 107 lines of *Arabidopsis* with the 250K array to determine which SNPs were polymorphic between two given lines to a high degree of confidence, using quality ensuring measures such as multiple prior data sets for sequence confirmation, the filtering of bad arrays and bad SNPs using mismatch rates, and removing non-binary SNPs. The Nordborg lab of USC, in collaboration with the Borevitz lab of U. Chicago, has since increased the number of genotyped accessions to approximately 1,000, using the same measures to ensure quality; this data is publicly available at http://walnut.usc.edu/2010. The ATTILE1R interrogates 1,683,620 unique genome locations with 25mer oligonucleotides distributed across the Arabidopis genome at an average spacing of ∼35 bp [15].

### Parent Sample Data

Li et al. hybridized the genomic DNA of parental lines of *Arabidopsis*, including Col-0 and Kr-0, to 250K ATSNPTILE1 arrays [13]. For each SNP there are four signals: the sense and antisense probe for the Col-0 genotype allele and the sense and antisense probe for the alternate ecotype allele. We spatially corrected these parent files [3], partitioned the data into sense and antisense signals, and took the difference in signal intensity between the reference allele probe and ecotype allele probe in both the sense and antisense sets.

### Simulations

We modified the method of Wolyn *et al*. [11] to produce simulations of XAM using the 250K ATSNPTILE1 arrays. We constructed simulations for populations of 100 RILs, 200 F_2_’s, and 1000 F_2_’s. We considered the same eight genetic models as Wolyn *et al*. [11] Five models considered a single QTL with various positions, additive effects, and dominance effects. Three models considered two QTL simultaneously, including two unlinked with minor additive effects, two linked in repulsion with major additive effects, and two unlinked exhibiting epistasis. The method accounted for variation due to differences in recombination, as well as phenotypic variation for each model. In accounting for phenotypic variation, we considered multiple measurements per RIL (n = 3). Using these simulated phenotypes, we selected two pools of plants representing the extreme 10 or 30% of phenotypic variation.

Since our method of BSA lacks the scaling step of Wolyn *et al*., rather than apply a mean feature intensity to normal noise for each probe, we took random selections of SNP intensity signals from actual hybridizations of parental accessions in the parent sample data. We randomly selected marker probe intensities from the reference genotype parent hybridization (i.e. the Col-0 array) and assigned them to the simulated genotypes that were homozygous for the reference genotype. We then randomly selected marker probe intensities in a hybridization from the outcross ecotype (in our simulations, Kr-0) and assigned them to the simulated genotypes homozygous for the outcross ecotype. Finally, we took the probe-wise mean of the parent Col-0 and Kr-0 intensities, and after observing that the results were distributed normally about zero, randomly selected these pseudo-F_1_ probe values and assigned them to the simulated heterozygous loci, thereby constructing a full simulation of SNP genotyping using our sample parent data. Because of the high degree of correlation between the sense and antisense probe signals, we used only the sense signals to produce the simulated genotype.

We then calculated each pool’s mean signal intensity for each probe, took the probe-wise difference between the mean signals, and applied a loess smooth (span = 0.25). Essentially, we performed BSA on the simulated SNP genotypes. For each permutation, we recorded the smoothed data’s maximum signal intensity and position of the maximum on each chromosome, as well as the minimum and position of the minimum on each chromosome.

We performed 1000 permutations for every combination of population, gene model, and selection intensity. Like Wolyn *et al*. we constructed horizontal thresholds for detection by considering the 95^th^ and 99^th^ percentile of the sorted maximum and minimum intensities on the unlinked chromosomes. These thresholds were calculated for each gene model, were observed to be approximately equal within each population/selection intensity group, then averaged for the final results. In gene models considering QTL on a single chromosome, four chromosomes were considered unlinked and used to construct thresholds, in models that considered two QTL on separate chromosomes simultaneously, the remaining three unlinked chromosomes were used to construct thresholds. We recorded a simulation as a failure if the maximum intensity on the linked chromosome(s) did not exceed our calculated threshold. Finally, we established 95% confidence intervals for the position on the peak signal at a modeled QTL by recording the central width along the linked chromosome that contained the maximum’s position in 950 of the 1000 simulations. Full summary data from the simulations and R scripts are available as supplemental files.

### BSA with SNPs

For our 250K SNP array data for BSA, we measured the elemental profile of 412 F_2_ plants from a cross between the Sulfur and Selenium mutant 78730 (in the Col-0 background) and the Ler-1 accession (Trays 1289–1290 and trays 1321–1323, data available at www.ionomicshub.org). Leaves from the 31 highest and 33 lowest S+Se accumulating plants (calculated as a percentage of the Col-0 accumulation in the same growth tray) were pooled and the genomic DNA was extracted using Qiagen kits. The DNA was sent to the University of Chicago array facility for hybridization to 250K SNP arrays and the Purdue Genomics Facility for hybridization to the ATTILE1R arrays. The CEL files have been deposited at GEO under accession GSE25509. All additional analysis was carried out using the R program (version 2.9.1) and the Bioconductor affy package (version 1.22.1). The CEL files were read in and spatially corrected using scripts from Borevitz et al. [3]. Using the data on polymorphism from the Nordborg lab, we selected SNPs that were known to be polymorphic between the two accessions. For both the sense and antisense probes, we first calculated the differences in signal intensity between the reference and alternate alleles for each SNP, and then subtracted the differences in one array from the other. After applying a loess smooth (span = 0.25), the magnitude of these comparisons of differences indicated a difference in allele frequency. To perform the method of BSA with microarrays outlined in previous studies [2]–[5], we adapted the scripts written by Borevitz *et al.* [3] and Wolyn *et al*. [11] to use our new SNP comparisons of differences as markers for difference in allele frequency. Data and R scripts are available at http://ars.usda.gov/mwa/bsasnp, the scripts are available as Files S1 and S2.

## Results and Discussion

### Accession Sample Data

**Figure 1:**
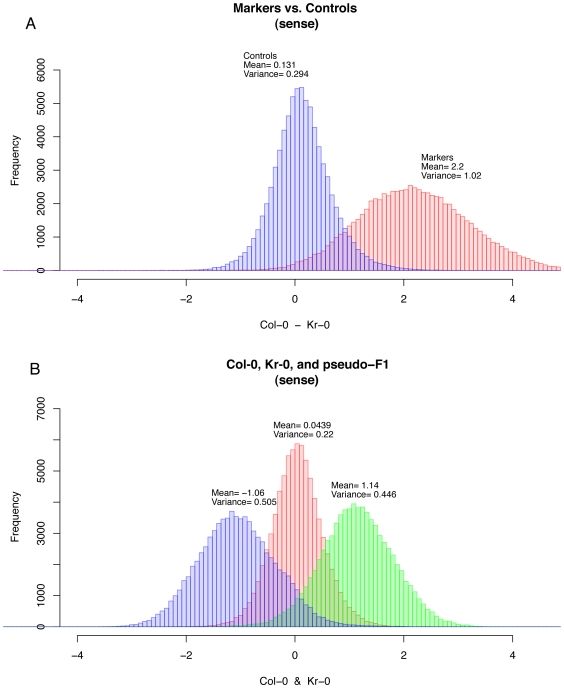
Probe signal distributions. (Top) Histograms of the difference in allele signals (Col-0 allele probe – other allele probe) of sense strand signals between the Col-0 and Kr-0 parent arrays for probe sets marked by the Atwell et al. [12] as polymorphic (markers) and not marked as polymorphic (controls). (Bottom) Histograms of the allele signals from the parent arrays and the pseudo-F_1_ array constructed from the mean of the parent arrays. The pseudo-F_1_ array signals are distributed about zero, as would be expected for an actual heterozygous plant.

To investigate the signal strength and noise levels inherent in the SNP platform, we analyzed hybridizations of genomic DNA from natural inbred accessions. While the signal from an array used for SFP measurements is simply the intensity of hybridization to a single probe, the signal from a SNP array is the difference in hybridization between the probe with allele 1 at the central base and the probe with allele 2 at the central base. The ATSNPTILE array was designed with reference to the Col-0 sequence, so each SNP set has probes for the Col-0 allele and the alleles are compared so that preferential hybridization to theCol-0 allele will result in a positive signal and preferential hybridization to the other allele will result in a negative signal. The signals from two different hybridizations produces the signal that can be used for BSA or XAM mapping, and the parent hybridizations represent the strongest possible signal (if both pools were homozygous for the respective parent at a given genomic region). For four different sets of accessions, we measured the difference in hybridization at the marker probes, those predicted by the Nordborg data set [12] to be polymorphic between the two parent accessions, and the control probes, those where the parents have the same allele and the difference should be ∼0 (Figure 1 and Figures S1, S2, S3, S4, S5, S6, S7). Although there are overlaps between the distributions of hybridization differences of the control and marker probes (Fig. 1a) the peaks are clearly separated. At most markers, therefore, large differences inthe allelic composition of BSA pools will be detectable, and smoothing the signal over adjacent markers along a chromosome should reduce false negative results due to low signal. To estimate the signal from a heterozygote hybridization, we created a pseudo-F_1_ array by taking the mean of two parent hybridizations (Fig. 1b). The peak of the pseudo-F1 hybridization was centered close to zero, as expected.

### Simulations

**Figure 2:**
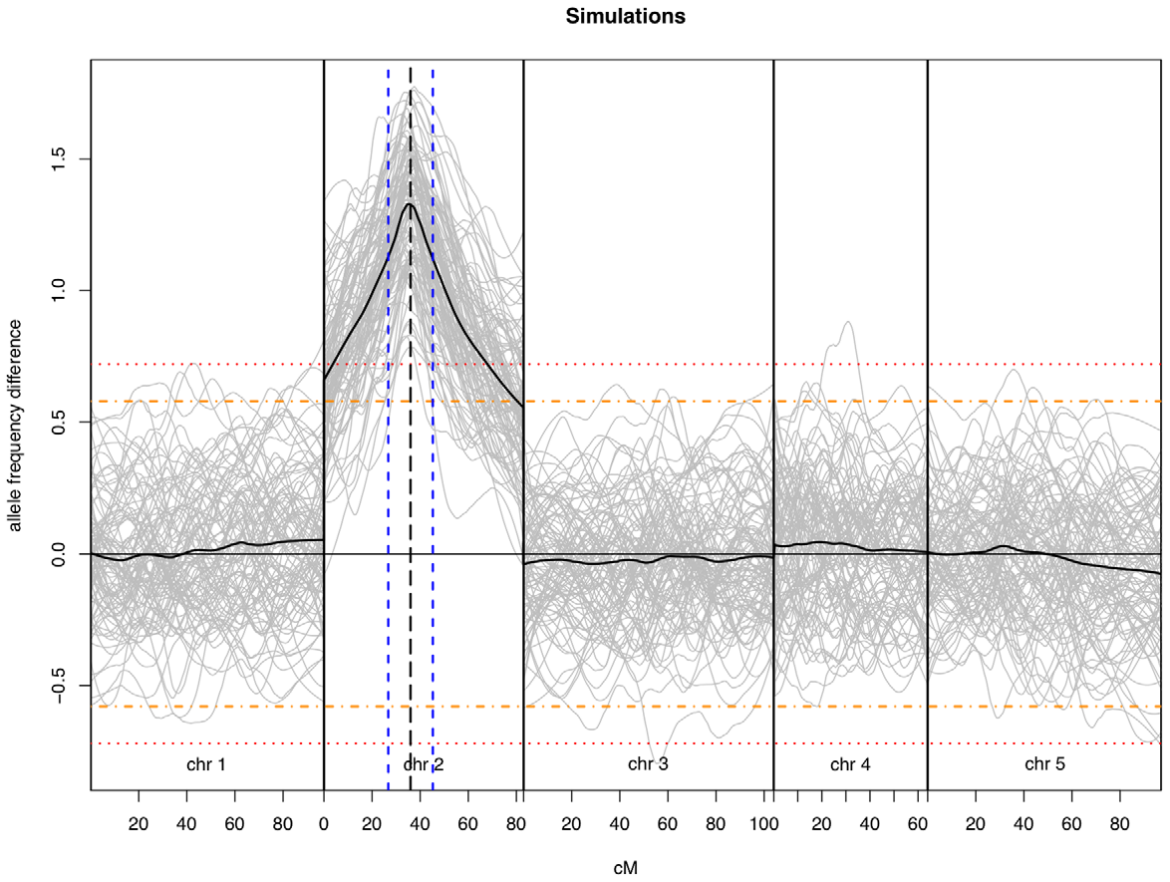
BSA using SNPs simulations. 100 simulations performed by selecting the extreme 10% of phenotypic variation for a population of 200 F_2_ plants with a major additive QTL at the 36^th^ centimorgan on the second chromosome, shown in gray. The bold lines represent the average of the 100 simulations for each chromosome. The dashed horizontal orange and red lines represent the 95 and 99% confidence thresholds for detection, respectively, established by the 1000 permutation simulations displayed in Table 1. The black dashed vertical line represents the location of the simulated QTL, with the neighboring blue dashed lines representing the boundaries of the 18.5 cM wide confidence interval formulated from our simulations.

In order to determine the feasibility of using the SNP array for BSA and XAM mapping, we simulated mapping experiments while varying the population (100 RILs, 200 and 1000 F2s) and bulk pool size (10% or 30%) and the underlying genetic model (eight models with either one or two loci varying in strength and interaction) (Table 1). For each scenario, we performed 1000 permutations and recorded the maximum and minimum difference in signal intensity for each chromosome between the simulated segregant pools, as well as the position of the maximum and minimum, to develop thresholds for detection and confidence intervals for position of the signal peak. Figure 2 illustrates the results from a simulated population of 200 F_2_ plants with a major additive QTL at the 36^th^ centimorgan on chromosome 2, selecting the extreme 10% of phenotypic variation as our bulks.

**Table 1:** Results of simulations.

***Population Type and Size***				**100 RILs**		**200 F_2_**		**1000 F_2_**	
***Selection Intensity***				10%	30%	10%	30%	10%	30%
***Confidence***				**Detection Thresholds**					
*95%*				1.18	0.7	0.58	0.34	0.27	0.15
*99%*				1.43	0.85	0.72	0.42	0.33	0.19
***Model***	***Effect***	***Chromosome No.***	***Position (cM)***	**Failure Rate (%)**					
1	Major Additive	Two	36	0.1	0	0	0	0	0
2	Major Dominant	Two	36	0	0	0	0	0	0
3	Moderate Additive	Two	36	12.5	4	10.4	3	0	0
4	Maj. Overdominance	Two	36	94.5	95.9	95.2	95.4	95.1	94.7
5	Major Additive	Two	2	0	0	0	0	0	0
6	Moderate Additive (unlinked)	Two	36	20.1	10.4	14.7	5.5	0	0
6	Moderate Additive (unlinked)	Five	41	17.4	7.7	12.2	4.4	0	0
7	Major Additive (linked in repulsion)	Two	36	24.1	42.3	55.5	48.9	0.7	0.5
7	Major Additive (linked in repulsion)	Two	56	29.8	45.1	58.5	52.6	0.9	0.2
8	Major Epistasis (unlinked)	Two	36	31.5	17.9	55.4	63.4	3	11.9
8	Major Epistasis (unlinked)	Five	41	32.6	15.7	58.7	65.3	3.9	12.3
***Model***	***Effect***	***Chromosome No.***	***Position (cM)***	**95% Confidence Interval (cM)**					
1	Major Additive	Two	36	10.4	5.6	18.5	9.4	3.8	3.2
2	Major Dominant	Two	36	11.2	5.4	20.5	14.4	4.3	3.6
3	Moderate Additive	Two	36	55.2	32.9	63.6	45.3	9.2	5.8
4	Maj. Overdominance	Two	36	83.0	83.0	83.0	83.0	83.0	83.0
5	Major Additive	Two	2	6.2	3.1	10.8	6.4	2.0	0.4
6	Moderate Additive (unlinked)	Two	36	60.1	52.4	66.1	61.0	10.2	6.7
6	Moderate Additive (unlinked)	Five	41	71.7	43.8	73.5	57.1	9.2	6.4
7	Major Additive (linked in repulsion)	Two	36	26.8	37.1	36.7	36.3	18.3	15.4
7	Major Additive (linked in repulsion)	Two	56	30.1	29.4	28.8	28.5	22.7	16.1
8	Major Epistasis (unlinked)	Two	36	77.2	58.2	83.0	83.0	42.6	71.3
8	Major Epistasis (unlinked)	Five	41	98.0	76.2	98.0	98.0	46.4	79.8

(Top) The horizontal detection thresholds calculated by considering the minimum signal not exceeded by the unlinked chromosomes in 95 and 99% of the simulations.

(Middle) The rate of failure, as a percentage, for a linked chromosome to exceed the 95% threshold for detection constructed from the maximum and minimum values on unlinked chromosomes.

(Bottom) The 95% confidence intervals constructed representing the central width of the chromosome that contained the peak in 950 of 1000 simulations. These data represent the precision of the mapping technique for each model, population, and selection intensity.

The results of our simulations exhibit the same patterns as the results of the simulations of Wolyn *et al.* [11]. Single major QTL were easily identified, with virtually no failures in any of the populations, while larger populations were required to ensure detection of all loci in more complex genetic models.

Two major loci linked in repulsion on the same chromosome (model 7) require large populations in order to obtain the requisite number of recombinations to separate them, while, as expected, this method is not suited for overdominant loci. The other two gene models exhibited relatively high failure rates and reduced precision with smaller population sizes. Accordingly, if researchers have any reason to believe that the trait under study is controlled by more than a single loci, they should err on the side of phenotyping more lines, and where complex genetic architecture is suspected, consider other mapping techniques. The results for our RIL populations tend to have a higher rate of failure and wider interval of confidence (i.e. are less precise) than the XAM simulations in Wolyn *et al*. [11], which can be attributed, at least in part, to the fact that our simulations considered 100 RILs with only 3 observations per RIL (n = 3), rather than 120 RILs with n = 10 in Wolyn *et al.*. This choice of settings for the simulations reflected experimental designs more likely for techniques such as ionomics where phenotyping in large numbers is more difficult than the hypocotyl length assay utilized in Wolyn *et al*.

### BSA with SNPs

**Figure 3:**
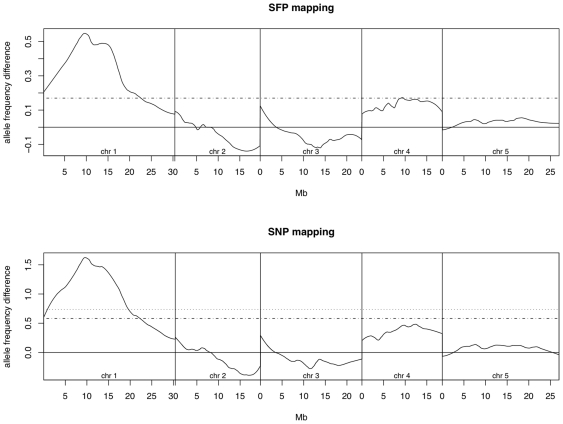
BSA using SNPs vs. SFPs. A comparison of BSA with SFP genotyping vs. BSA with SNP genotyping using the same genomic DNA for hybridization. A. The dashed line represents the detection threshold of 0.17 established by Borevitz *et al*. [3]. B The dashed lines represent the 95 and 99% confidence thresholds for detection established by our simulations.

To test the SNP BSA method on real samples, we performed BSA analysis on a population of F2 plants derived from a cross of Ler-0 to the high Sulfur and Selenium ionomics mutant 78730 (an EMS mutant in the Col-0 background). Lines were scored for their percentage change in each element compared to Col-0 grown in the same tray. 31 plants were identified as unambigiously mutant and used as the mutant bulk, while 33 with the lowest Sulphur/Selenium phenotype were used for the control pool. Genomic DNA was isolated from both pools and hybridized to either the ATTILE1R or ATSNPTILE arrays. BSA mapping was performed according to the protocols of this paper (ATSNPTILE) or the protocols of Borevitz et al [3] (using previosuly obtained hybridizations of Col-0 and Ler-0. The BSA traces produced by both methods are highly correlated (Fig. 3), indicating that the methods are producing similar results, and identified a region centered around 10 Mb on Chr 1 as the location of the causal locus. PCR analysis of known Col-0/Ler-0 markers located 9.2 Mb and 11 Mb on Chr 1 confrimed the BSA predicted location and fine mapping of the locus is ongoing. Given that the cost of the ATSNPTILE array is ∼1/2 that of the ATTILE1 (and the older ATH1 array), and SNP mapping doesn’t require parent hybs for marker detection, the SNP array is clearly the most economical option. In an effort to make this analysis more accessible to researchers with limited bioinformatics experience, we have created scripts that query the user for appropriate variables at the relevant steps which are available as supplemental data files and at the website: http://ars.usda.gov/mwa/bsasnp.

The mapping performed on the 78730 mutant used a subset of the available markers on both arrays: 30,000 SFP markers were used, although, permutation testing suggests that 100,000 SFPs could be used with a false discovery rate of 0.05. The SNP mapping used a randomly selected set of 1/4^th^ of the ∼70,000 SNPs available in most parent combinations, and only used the sense strand for the mapping. These marker densities are ∼10X what is needed for BSA or XAM mapping of most RIL and F2 populations, but may be useful for applications that require finer mapping, such as BSA mapping on pools derived from lines with known breakpoints around a candidate loci or mapping of break points in RIL lines [16], [17].

### Conclusions

Here we have demonstrated that the ATSNPTILE array is well suited for BSA and XAM mapping and represents a low cost alternative to the arrays previously used for SFP mapping. We have performed simulations that demonstrate that this method can easily detect the causal region when a trait is controlled by a single loci, while more complex genetic scenarios will require larger populations to ensure detection.

## Supporting Information

Figure S1 **Kr-0, antisense probe distributions.** (Top) Histograms of the antisense probe-wise difference in allele signals between the Col-0 and Kr-0 parent arrays for probe sets marked by the Atwell et al. [12] as polymorphic (markers) and not marked as polymorphic (controls). (Bottom) Histograms of the allele signals from the parent arrays and the pseudo-F_1_ array constructed from the mean of the parent arrays.
(PDF)

Figure S2 **Eden-1, antisense probe distributions.** (Top) Histograms of the antisense probe-wise difference in allele signals between the Col-0 and Eden-1 parent arrays for probe sets marked by the Atwell et al. [12] as polymorphic (markers) and not marked as polymorphic (controls). (Bottom) Histograms of the allele signals from the parent arrays and the pseudo-F_1_ array constructed from the mean of the parent arrays.
(PDF)

Figure S3 **Eden-1, sense probe distributions.** (Top) Histograms of the sense probe-wise difference in allele signals between the Col-0 and Eden-1 parent arrays for probe sets marked by the Atwell et al. [12] as polymorphic (markers) and not marked as polymorphic (controls). (Bottom) Histograms of the allele signals from the parent arrays and the pseudo-F_1_ array constructed from the mean of the parent arrays.
(PDF)

Figure S4 **Van-0, antisense probe distributions.** (Top) Histograms of the antisense probe-wise difference in allele signals between the Col-0 and Van-0 parent arrays for probe sets marked by the Atwell et al. [12] as polymorphic (markers) and not marked as polymorphic (controls). (Bottom) Histograms of the allele signals from the parent arrays and the pseudo-F_1_ array constructed from the mean of the parent arrays.
(PDF)

Figure S5 **Van-0, sense probe distributions.** (Top) Histograms of the sense probe-wise difference in allele signals between the Col-0 and Van-0 parent arrays for probe sets marked by the Atwell et al. [12] as polymorphic (markers) and not marked as polymorphic (controls). (Bottom) Histograms of the allele signals from the parent arrays and the pseudo-F_1_ array constructed from the mean of the parent arrays.
(PDF)

Figure S6 **Ler-1, antisense probe distributions.** (Top) Histograms of the antisense probe-wise difference in allele signals between the Col-0 and Ler-1 parent arrays for probe sets marked by the Atwell et al. [12] as polymorphic (markers) and not marked as polymorphic (controls). (Bottom) Histograms of the allele signals from the parent arrays and the pseudo-F_1_ array constructed from the mean of the parent arrays.
(PDF)

Figure S7 **Ler-1, sense probe distributions.** (Top) Histograms of the sense probe-wise difference in allele signals between the Col-0 and Ler-1 parent arrays for probe sets marked by the Atwell et al. [12] as polymorphic (markers) and not marked as polymorphic (controls). (Bottom) Histograms of the allele signals from the parent arrays and the pseudo-F_1_ array constructed from the mean of the parent arrays.
(PDF)

File S1 **BSA using SNPs supplies script.** R script containing the functions necessary to perform BSA using the ATSNPTILE1 array. This script supplies information for File S2. This is not the script to open in order to perform BSA using the ATPSNPTILE1 array, but is needed for the process.
(TXT)

File S2 **BSA using SNPs script.** R script that contains first instructions and relies on the information in File S1 to perform BSA using the ATPSNPTILE1 array. This is the script to open to perform our method.
(TXT)
