# Supplementary figures and images for: Bulk Segregant Analysis Using Single Nucleotide Polymorphism Microarrays
Source: PLoS One. 2011 Jan 27;6(1):e15993. doi: 10.1371/journal.pone.0015993 (PMC3029305; doi:10.1371/journal.pone.0015993)

## Markers vs. Controls (antisense)

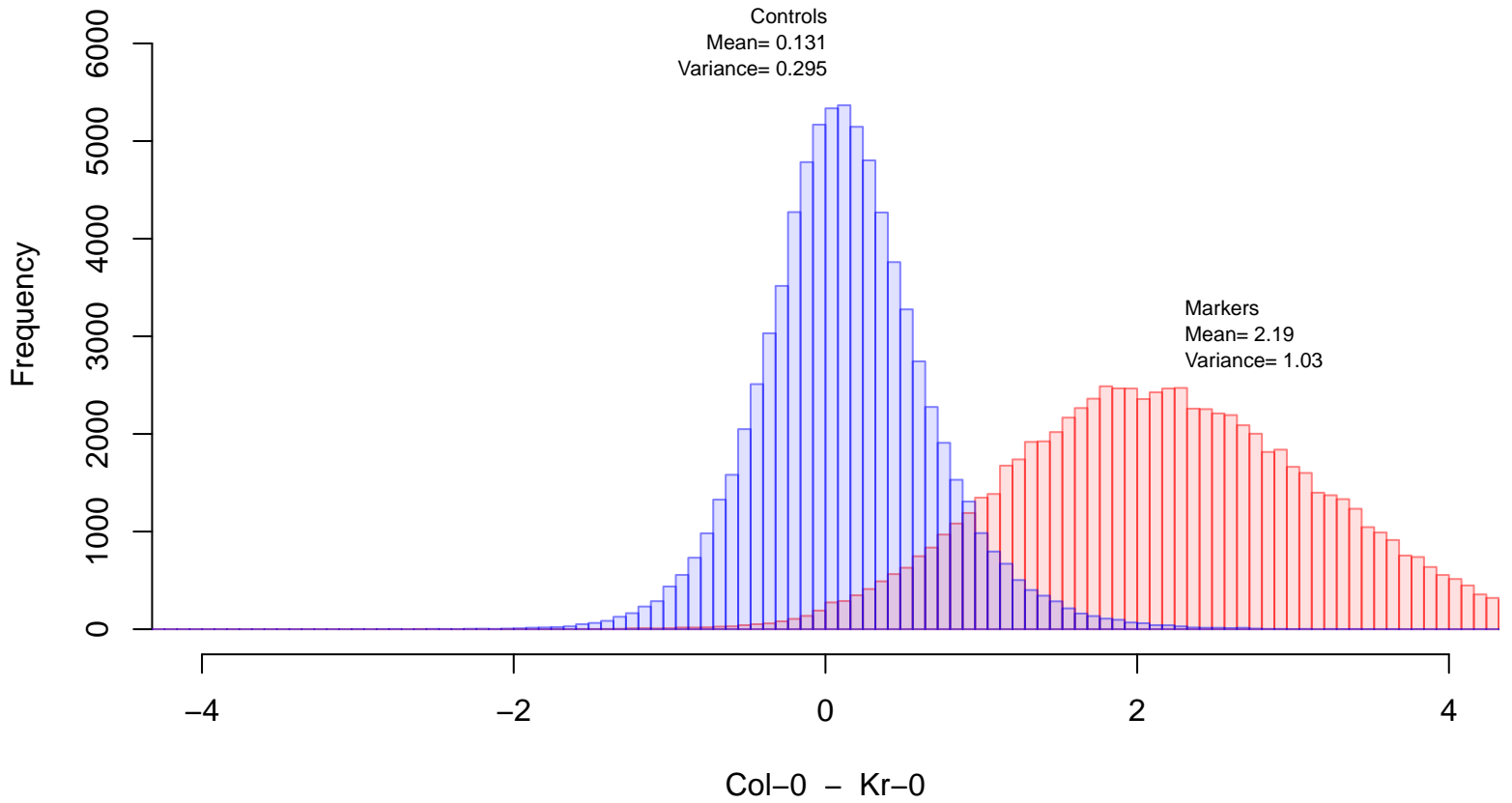

## P1, P2, and pseudo-F1 (antisense)

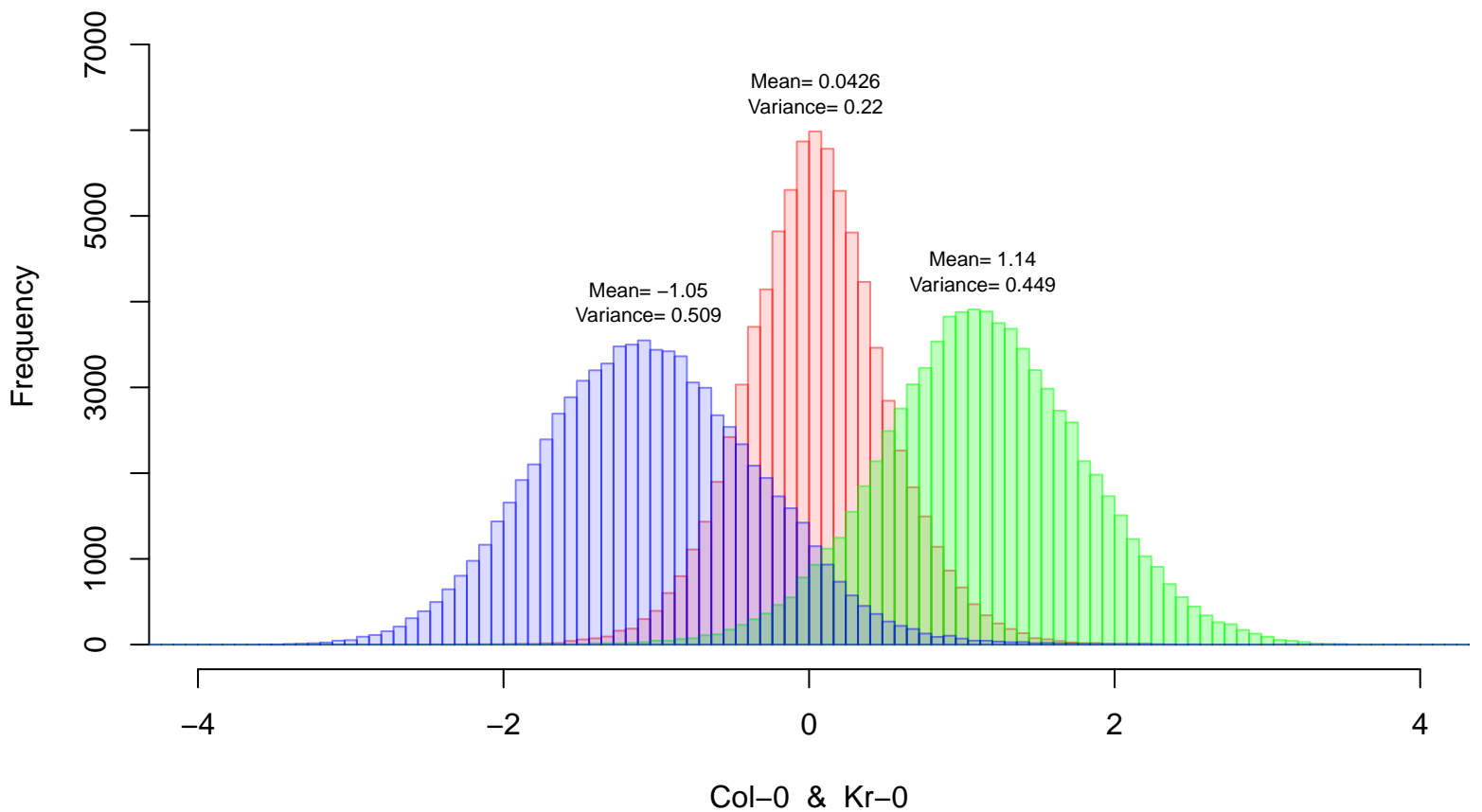

Supplement: Figure S1 — Kr-0, antisense probe distributions. (Top) Histograms of the antisense probe-wise difference in allele signals between the Col-0 and Kr-0 parent arrays for probe sets marked by the Atwell et al. [12] as polymorphic (markers) and not marked as polymorphic (controls). (Bottom) Histograms of the allele signals from the parent arrays and the pseudo-F1 array constructed from the mean of the parent arrays. (PDF) [file pone.0015993.s001.pdf]

## Markers vs. Controls (antisense)

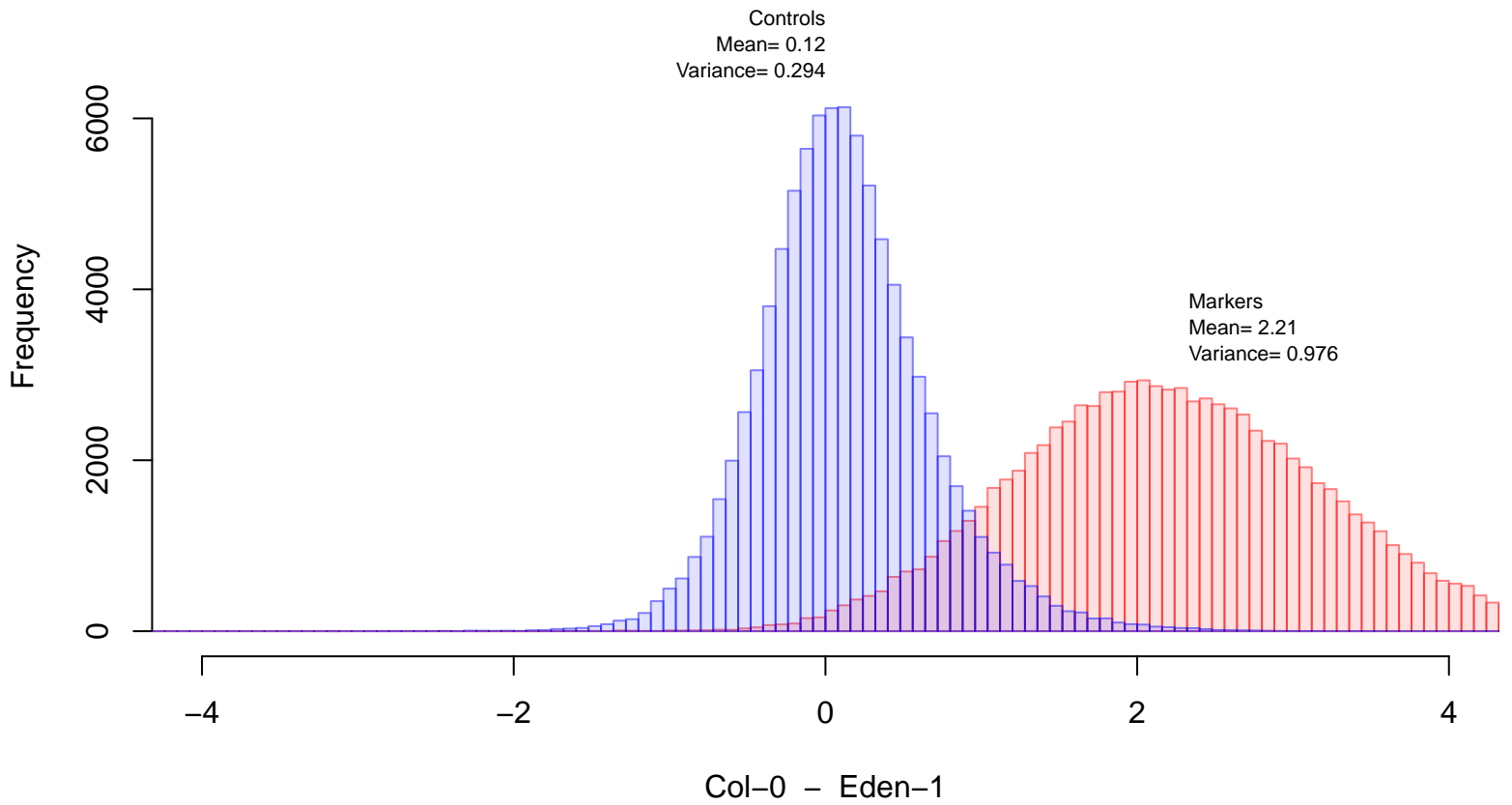

## P1, P2, and pseudo-F1 (antisense)

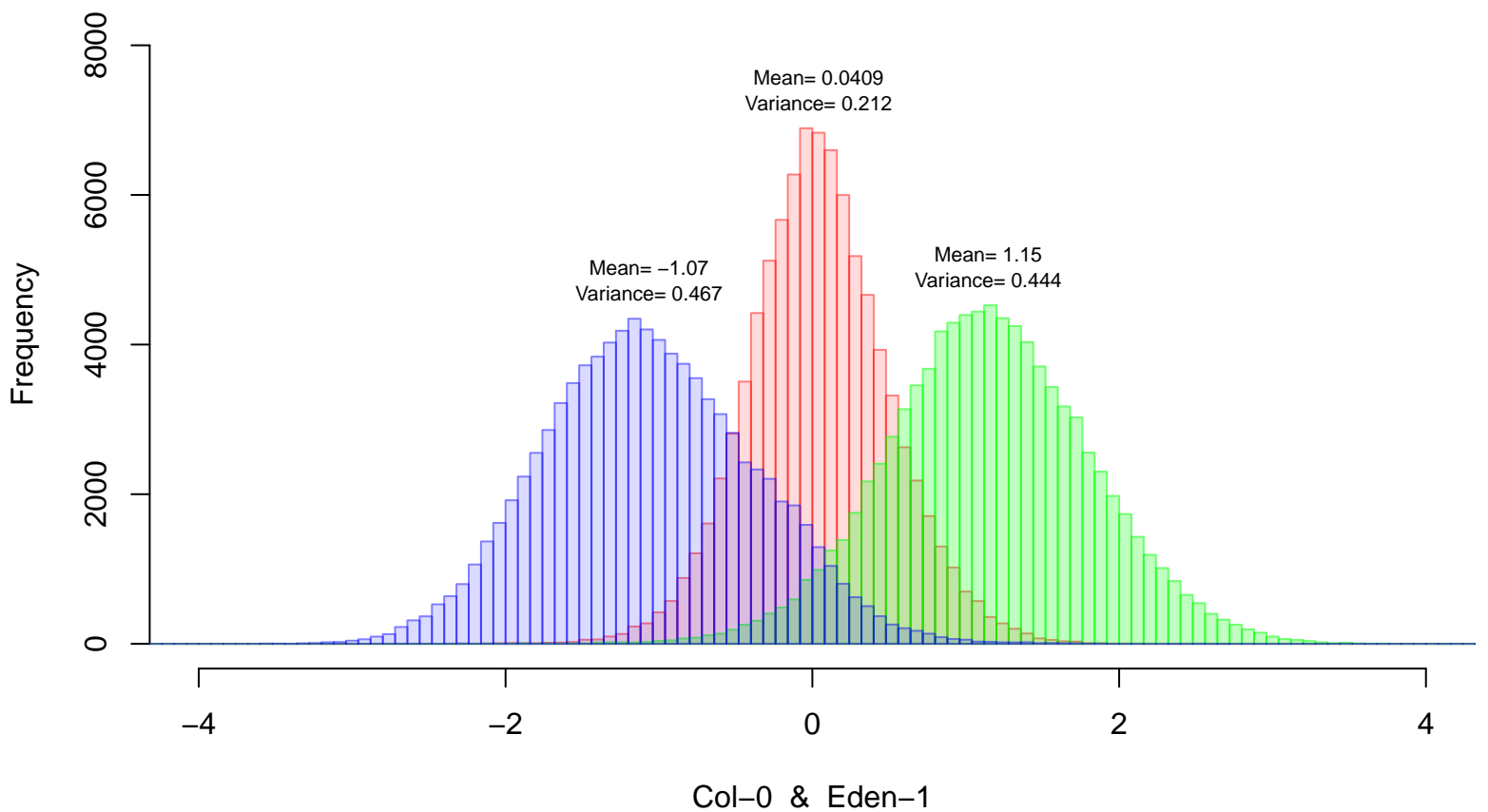

Supplement: Figure S2 — Eden-1, antisense probe distributions. (Top) Histograms of the antisense probe-wise difference in allele signals between the Col-0 and Eden-1 parent arrays for probe sets marked by the Atwell et al. [12] as polymorphic (markers) and not marked as polymorphic (controls). (Bottom) Histograms of the allele signals from the parent arrays and the pseudo-F1 array constructed from the mean of the parent arrays. (PDF) [file pone.0015993.s002.pdf]

## Markers vs. Controls (sense)

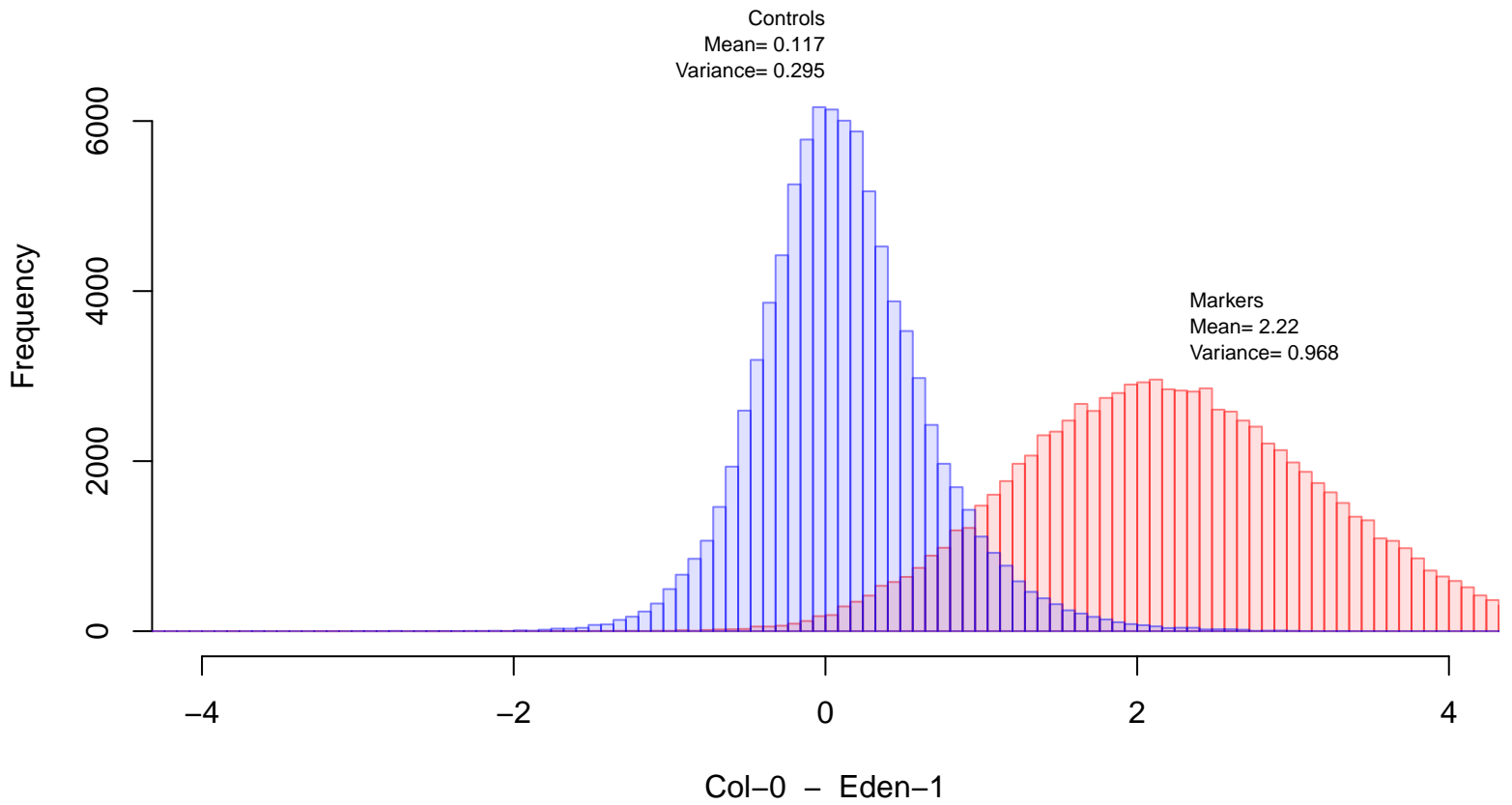

## P1, P2, and pseudo-F1 (sense)

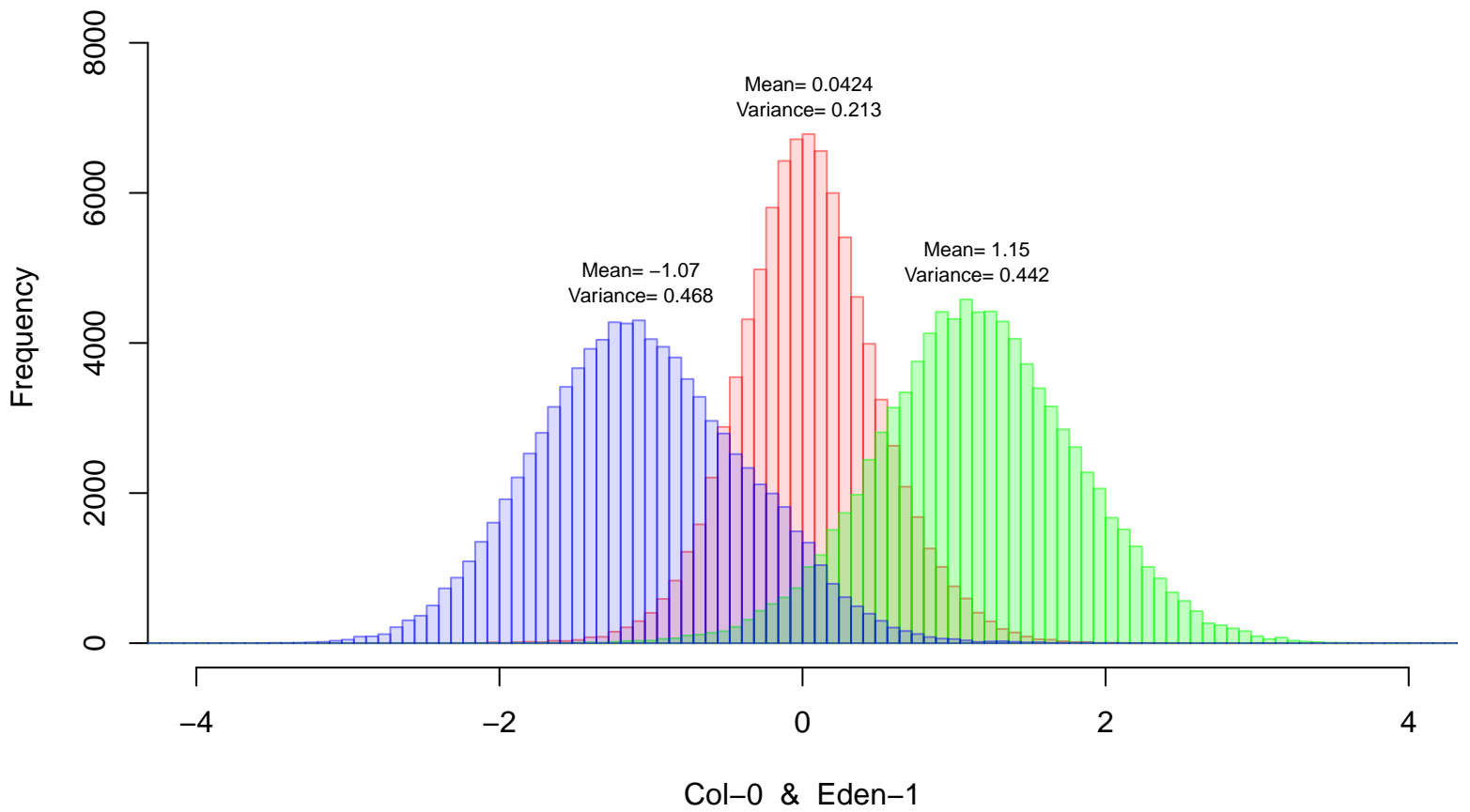

Supplement: Figure S3 — Eden-1, sense probe distributions. (Top) Histograms of the sense probe-wise difference in allele signals between the Col-0 and Eden-1 parent arrays for probe sets marked by the Atwell et al. [12] as polymorphic (markers) and not marked as polymorphic (controls). (Bottom) Histograms of the allele signals from the parent arrays and the pseudo-F1 array constructed from the mean of the parent arrays. (PDF) [file pone.0015993.s003.pdf]

## Markers vs. Controls (antisense)

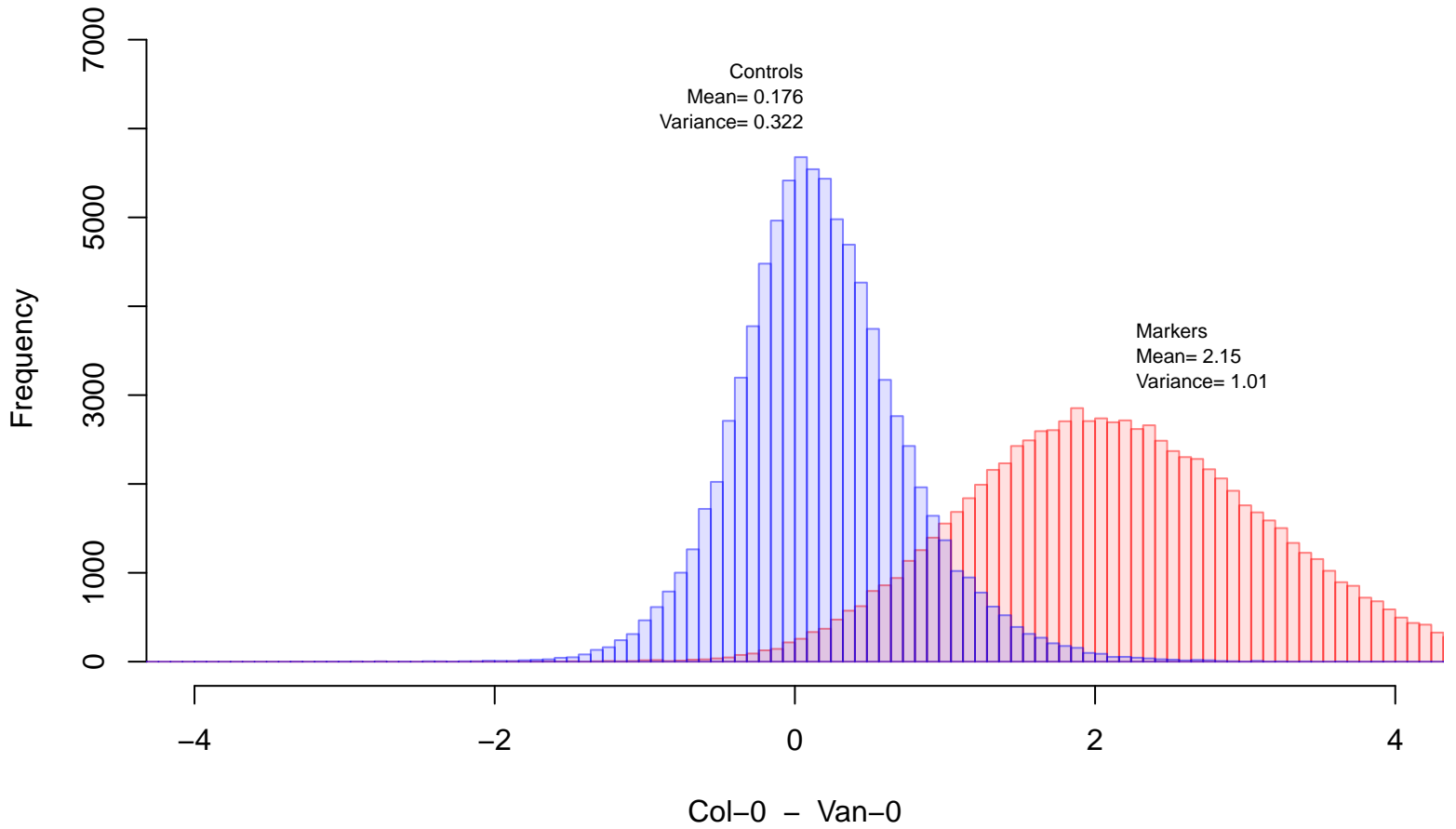

## P1, P2, and pseudo-F1 (antisense)

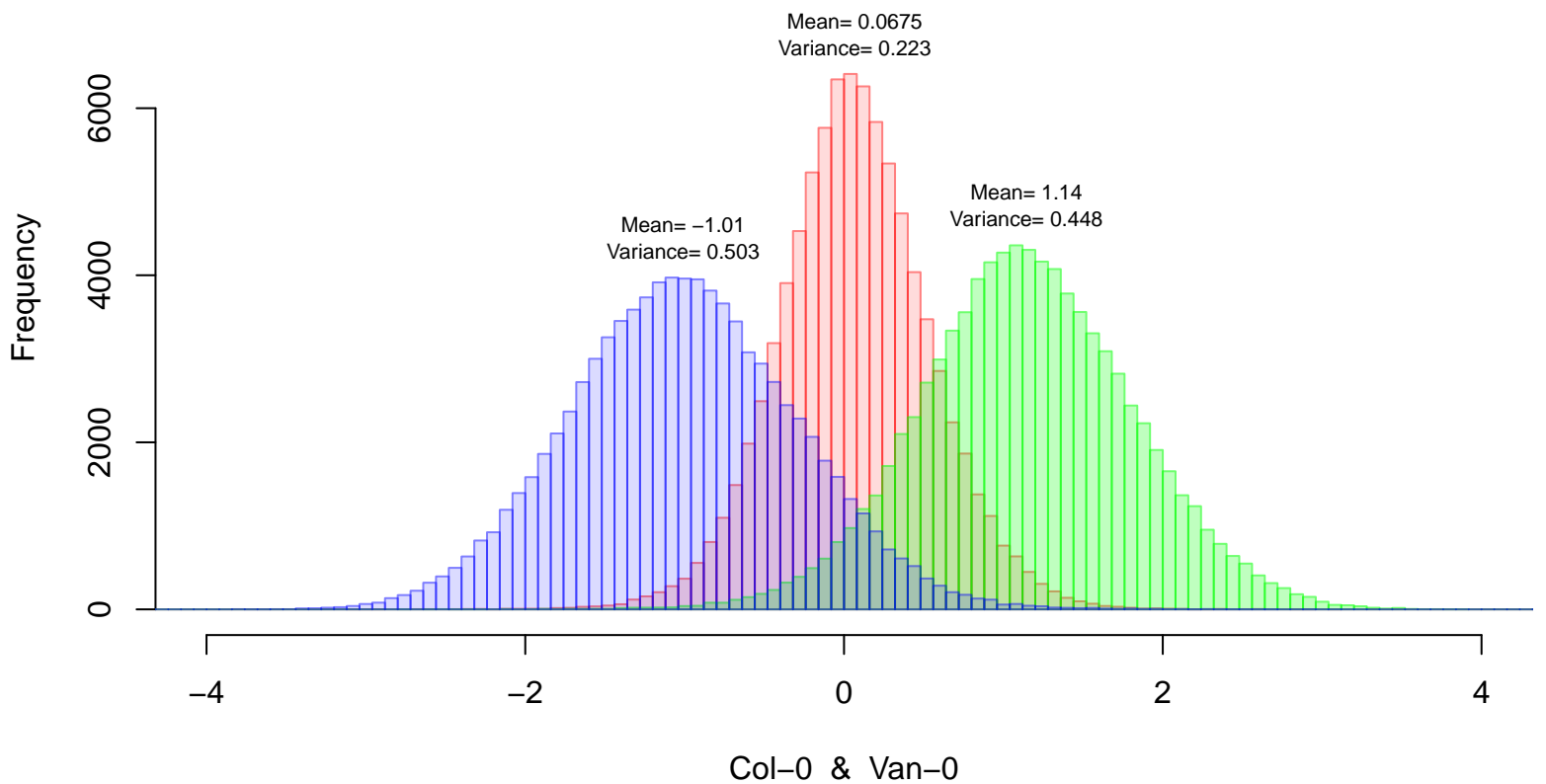

Supplement: Figure S4 — Van-0, antisense probe distributions. (Top) Histograms of the antisense probe-wise difference in allele signals between the Col-0 and Van-0 parent arrays for probe sets marked by the Atwell et al. [12] as polymorphic (markers) and not marked as polymorphic (controls). (Bottom) Histograms of the allele signals from the parent arrays and the pseudo-F1 array constructed from the mean of the parent arrays. (PDF) [file pone.0015993.s004.pdf]

## Markers vs. Controls (sense)

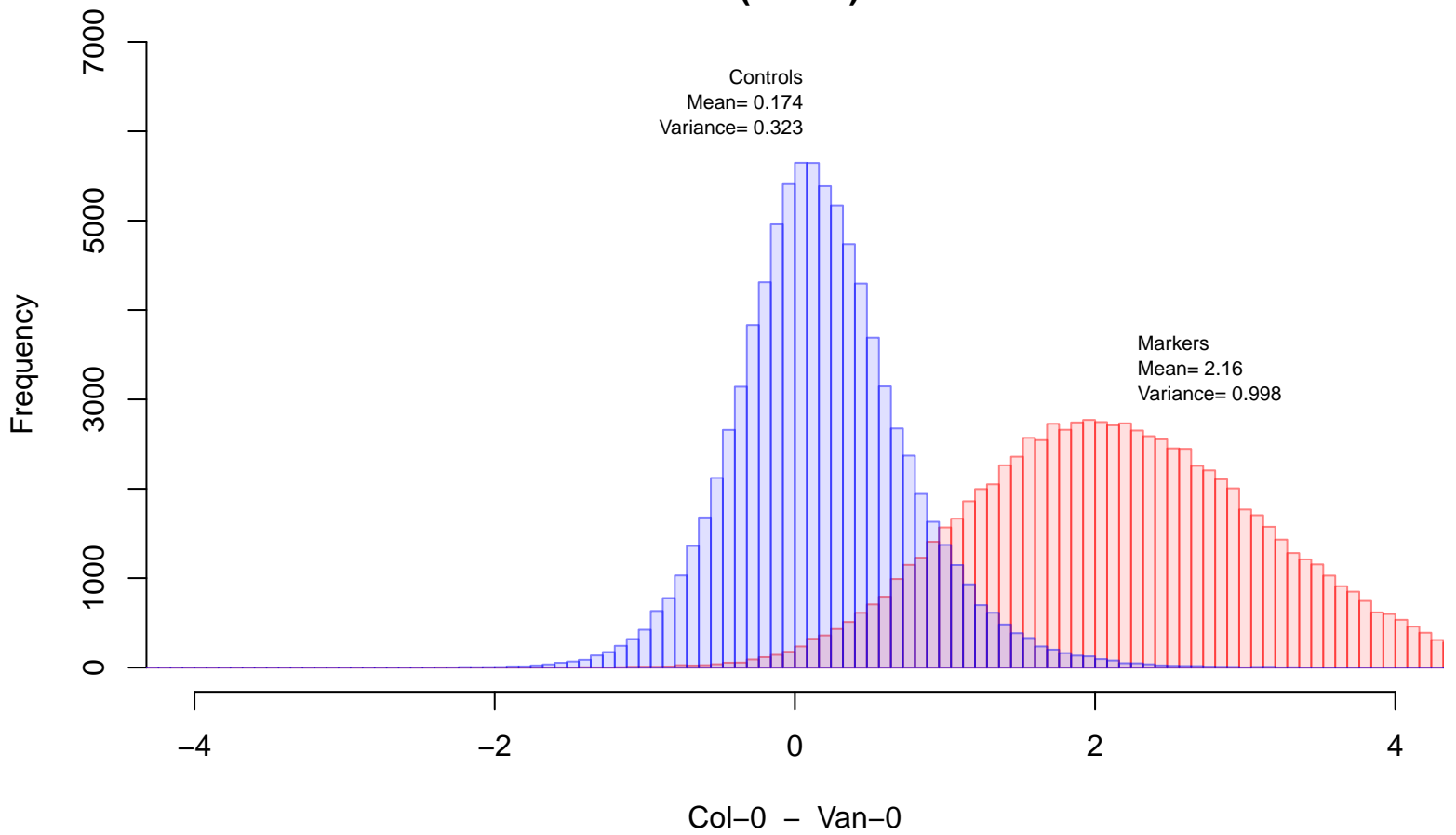

## P1, P2, and pseudo-F1 (sense)

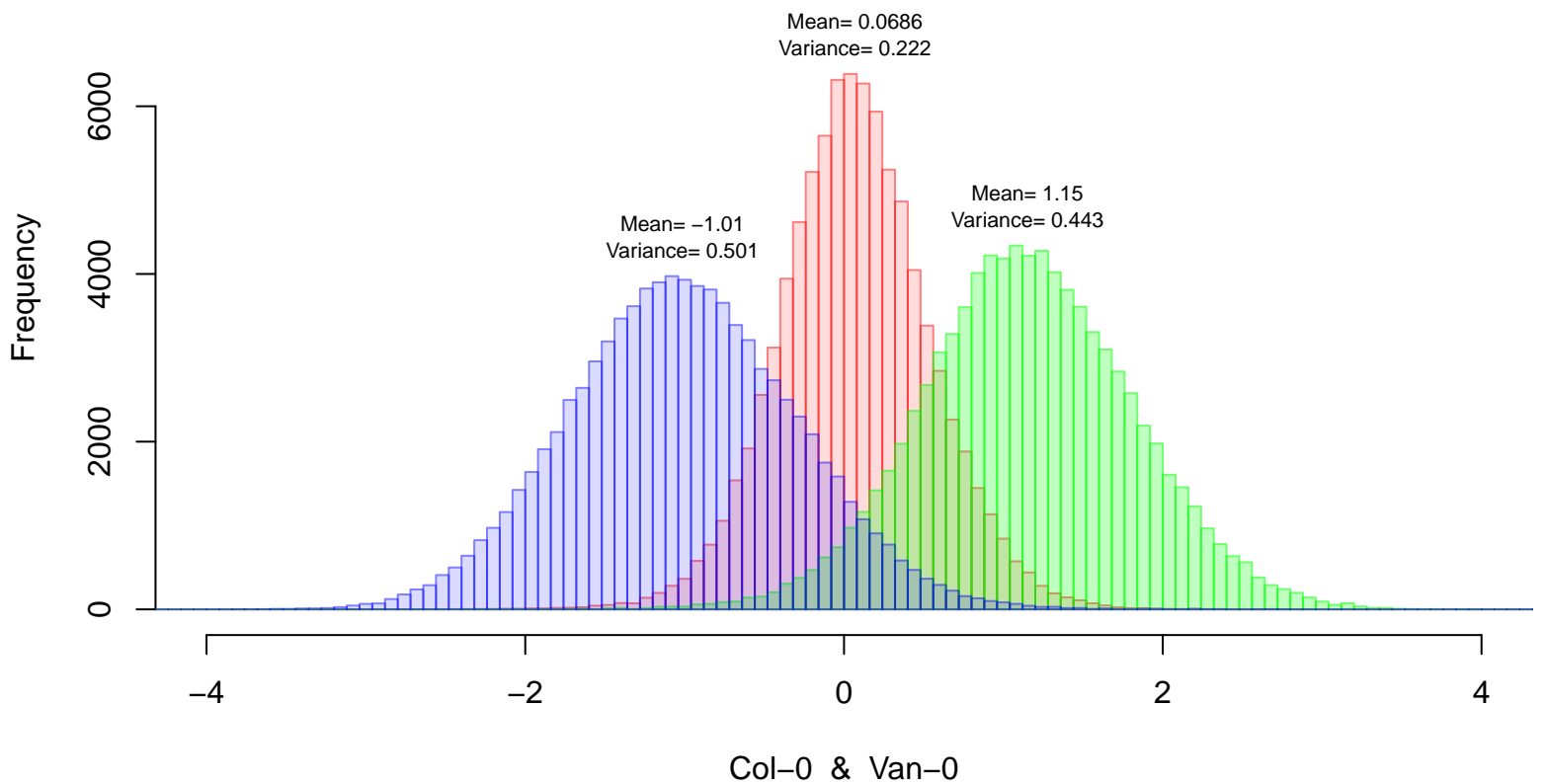

Supplement: Figure S5 — Van-0, sense probe distributions. (Top) Histograms of the sense probe-wise difference in allele signals between the Col-0 and Van-0 parent arrays for probe sets marked by the Atwell et al. [12] as polymorphic (markers) and not marked as polymorphic (controls). (Bottom) Histograms of the allele signals from the parent arrays and the pseudo-F1 array constructed from the mean of the parent arrays. (PDF) [file pone.0015993.s005.pdf]

## Markers vs. Controls (antisense)

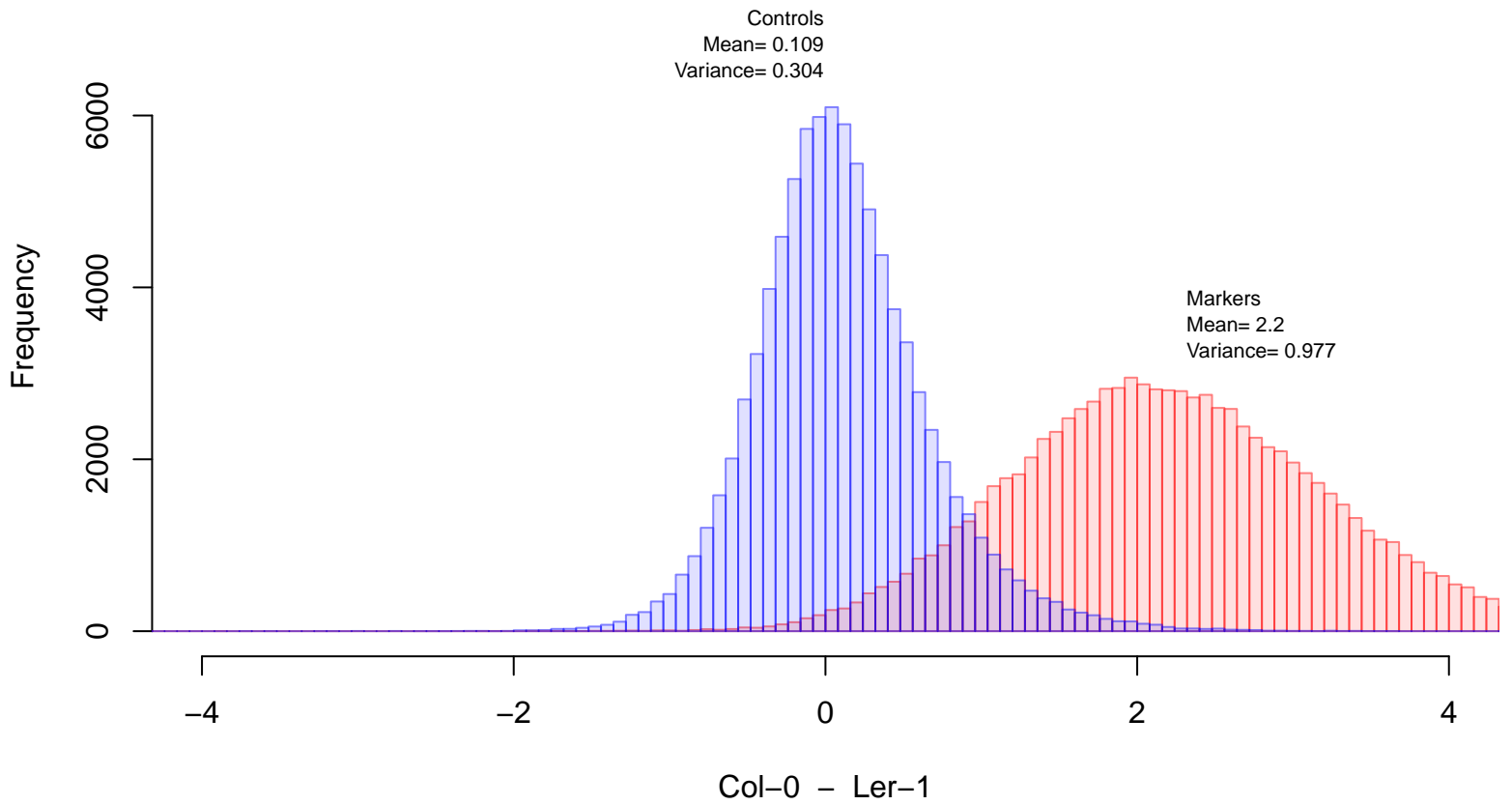

## P1, P2, and pseudo-F1 (antisense)

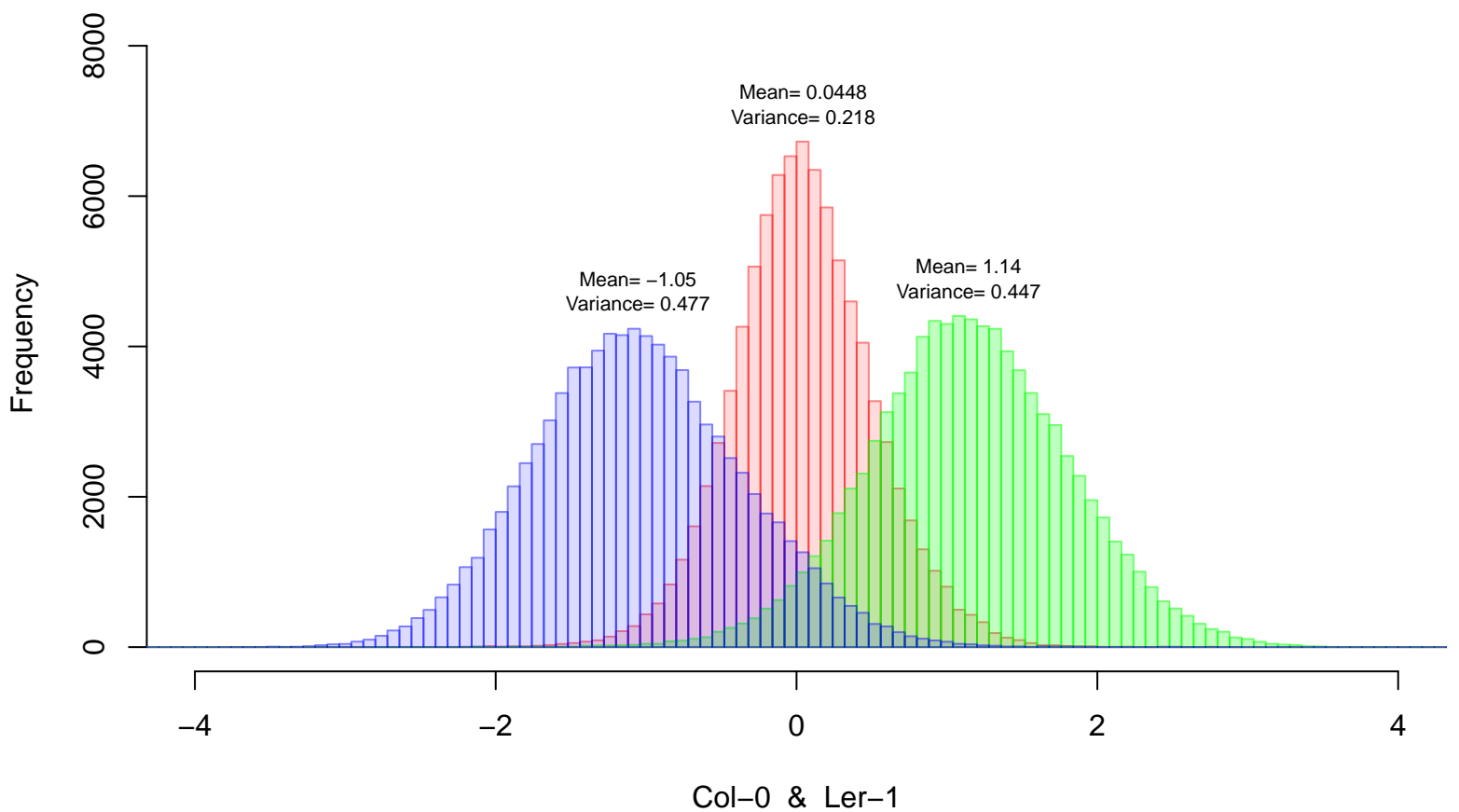

Supplement: Figure S6 — Ler-1, antisense probe distributions. (Top) Histograms of the antisense probe-wise difference in allele signals between the Col-0 and Ler-1 parent arrays for probe sets marked by the Atwell et al. [12] as polymorphic (markers) and not marked as polymorphic (controls). (Bottom) Histograms of the allele signals from the parent arrays and the pseudo-F1 array constructed from the mean of the parent arrays. (PDF) [file pone.0015993.s006.pdf]

## Markers vs. Controls (sense)

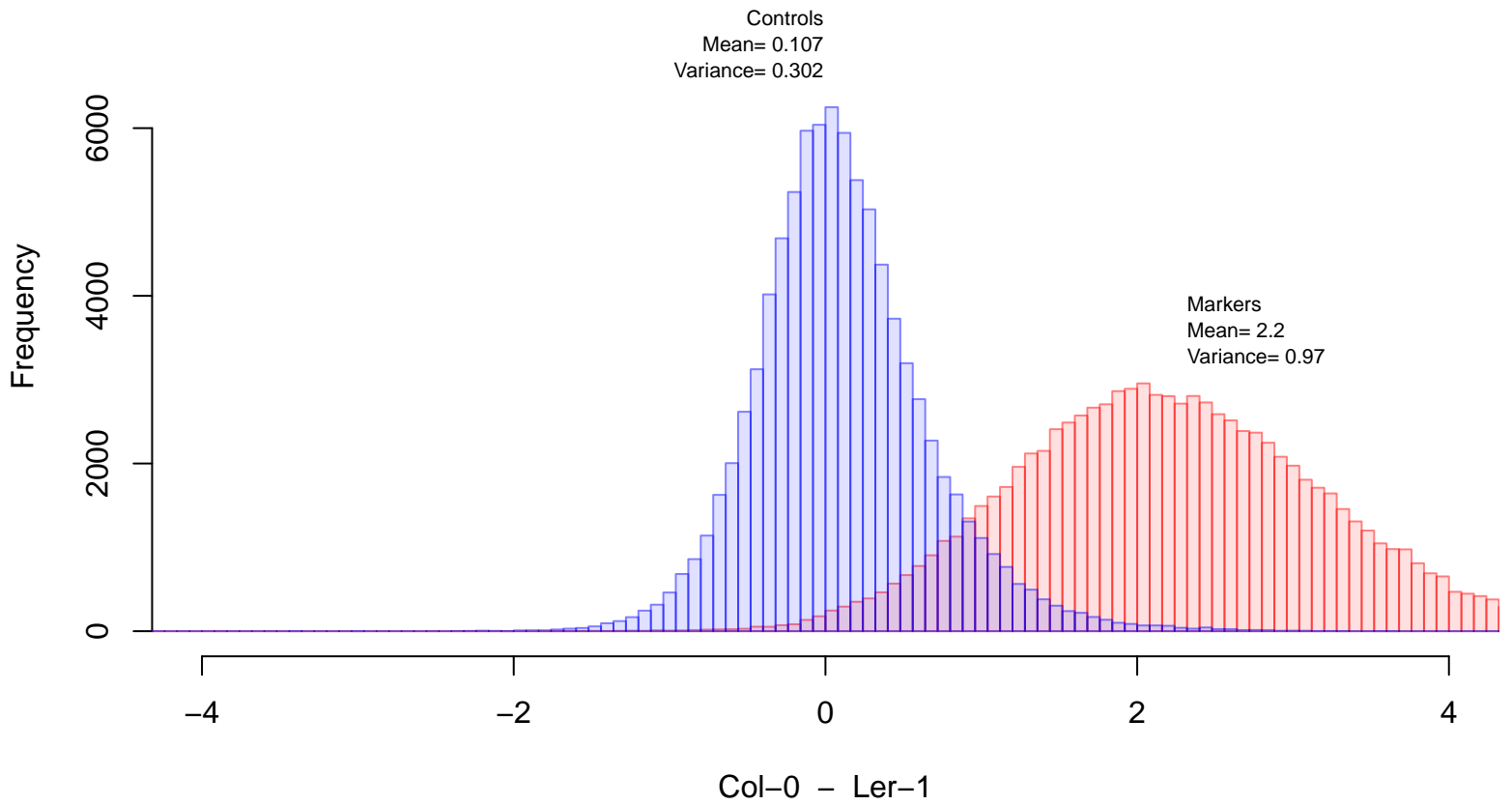

## P1, P2, and pseudo-F1 (sense)

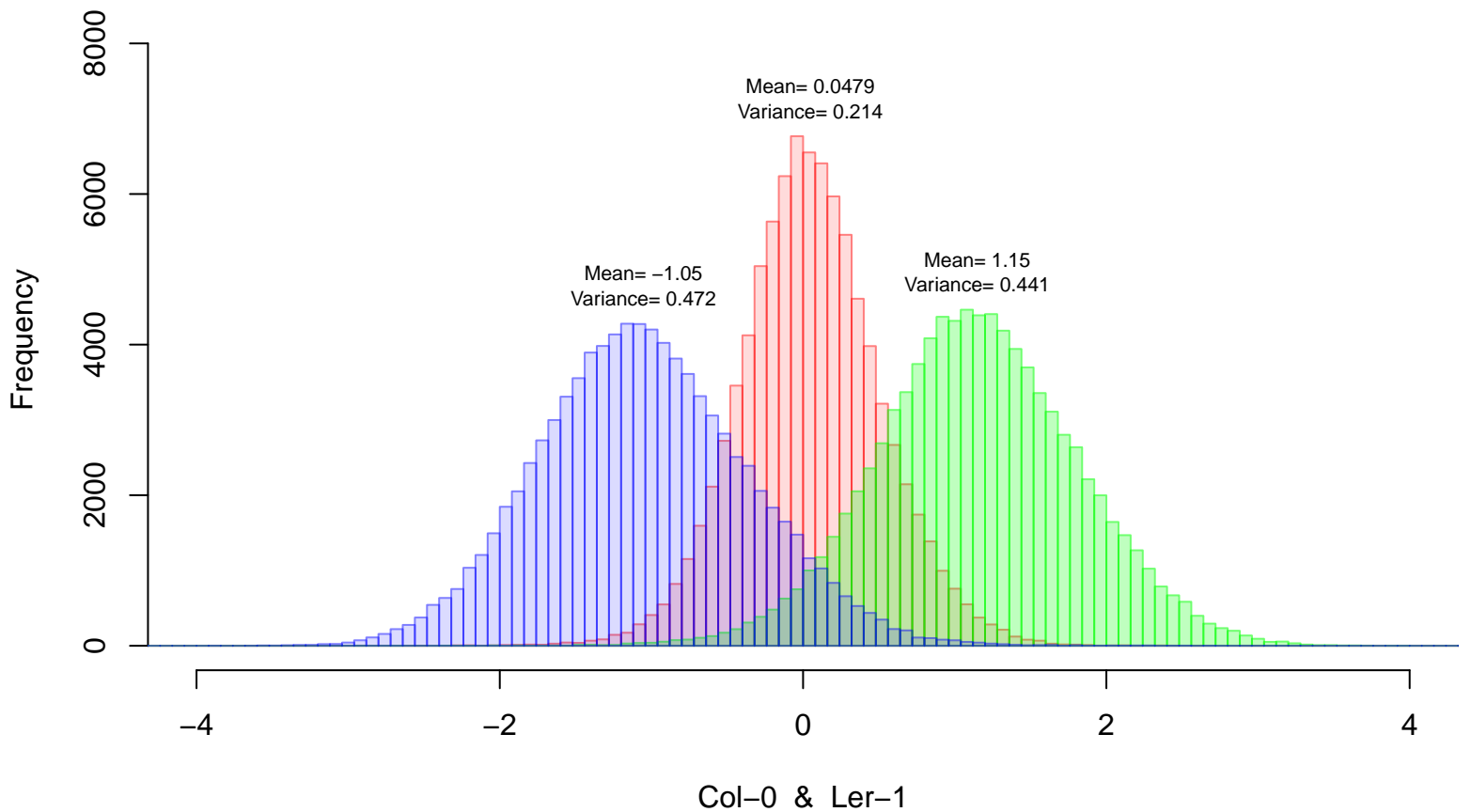

Supplement: Figure S7 — Ler-1, sense probe distributions. (Top) Histograms of the sense probe-wise difference in allele signals between the Col-0 and Ler-1 parent arrays for probe sets marked by the Atwell et al. [12] as polymorphic (markers) and not marked as polymorphic (controls). (Bottom) Histograms of the allele signals from the parent arrays and the pseudo-F1 array constructed from the mean of the parent arrays. (PDF) [file pone.0015993.s007.pdf]
